# Norcantharidin/Cu^2+^ dual-depleting GSH nanocatalyst with pH-responsive for CT/CDT synergistic cancer therapy

**DOI:** 10.1016/j.mtbio.2025.101959

**Published:** 2025-06-06

**Authors:** Xiaohuan Guo, Bingbing Cai, Qi Fang, Yanyan Chen, Yuzhu Zhou, Zhixing Liang, Changchun Wen, Yan-Cheng Liu, Hong Liang

**Affiliations:** State Key Laboratory for Chemistry and Molecular Engineering of Medicinal Resources, Key Laboratory for Chemistry and Molecular Engineering of Medicinal Resources (Ministry of Education of China), Guangxi Key Laboratory of Chemistry and Molecular Engineering of Medicinal Resources, School of Chemistry and Pharmaceutical Sciences, Guangxi Normal University, Guilin, 541004, China

**Keywords:** Norcantharidin, PEG, GSH depletion, pH-response, Synergistic therapy

## Abstract

The high level of glutathione (GSH) in tumor cells can consume reactive oxygen species (ROS), seriously affecting the efficacy of chemodynamic therapy (CDT). Although it took a great deal of effort, developing a tumor-specific CDT that efficiently depletes GSH remains a formidable challenge. Herein, we propose a pH-responsive nanocatalyst containing the active molecule norcantharidin (NCTD) and Cu^2+^ for dual GSH depletion, achieving efficient GSH depletion. Due to the weakly acidic tumor microenvironment (TME), the catalyst releases NCTD and Cu^2+^ in a pH-responsive manner for the synergistic therapy of chemotherapy (CT) and CDT. Both components consume GSH and subsequently produce ROS, reducing the antioxidant capacity of cancer cells while increasing oxidative stress. This disrupts cellular redox homeostasis, leading to mitochondrial dysfunction and inducing tumor cell apoptosis. This work not only develops nanomaterials with dual GSH depletion capabilities for high-efficiency CDT but also achieves synergistic CT and CDT tumor therapy with the addition of NCTD, an active ingredient of traditional Chinese medicine.

## Graphical abstract

**Figure:**
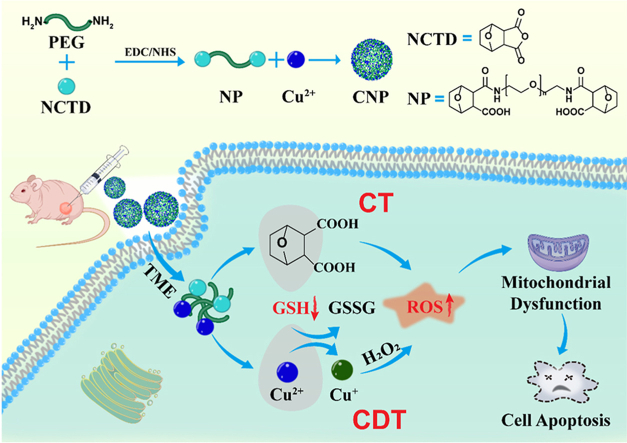

## 1 Introduction

Cancer characterized by its high recurrence rate, mortality, and propensity for metastasis, poses a significant threat to human health and life [1,2]. Although the widespread use of chemotherapy (CT) and radiotherapy in cancer treatment, substantial challenges remain in overcoming multidrug resistance, the intricate nature of tumor pathology, and preventing metastasis [3,4]. Moreover, the non-targeted nature of CT often leads to severe drug resistance and side effects [[5], [6], [7]]. In solid tumors, leveraging the permeability and retention effect [8], nanoparticle-based drug delivery systems can enhance targeting and uptake efficacy of CT drugs, thereby reducing their toxic side effects, with the advance of nanomedicine, numerous strategies have emerged for oncotherapy [[9], [10], [11]]. The rational design and functionalization of nanomaterials can further integrate multiple therapeutic approaches to reduce drug resistance and enhance the efficiency of tumor treatment [[12], [13], [14]].

Chemodynamic therapy (CDT) represents a novel nanotherapeutic approach that utilizes Fenton or Fenton-like reactions to transform excess H_2_O_2_ within the tumor microenvironment (TME) into cytotoxic hydroxyl radicals (·OH). This process can subsequently induce apoptosis in cancer cells [15,16]. The elevated glutathione (GSH) levels and lower pH in the TME create conditions more conducive to Fenton or Fenton-like reactions compared to normal tissues [17,18], which results in increased production of ·OH within tumor tissue. The generation of highly toxic reactive oxygen species (ROS), which is closely linked to the effectiveness of CDT, can inflict significant damage on cancer cells by disrupting the vascular system, damaging cell lipids and DNA, and inactivating proteins [[19], [20], [21]]. However, high levels of GSH within the tumor can clear the production of highly toxic ROS, reduce oxidative stress, and reduce therapeutic efficient [22]. Therefore, reducing the GSH levels within tumors is an effective approach to enhance the efficiency of CDT [[23], [24], [25]].

In comparison to classical Fe^2+^, Cu^+^ exhibit greater Fenton-like activity and a broader pH application range, and the high-priced copper ion (Cu^2+^) can be reduced by GSH to low-priced copper ion (Cu^+^) [26,27]. Along with the consumption of GSH, Cu^+^ ions catalyze the conversion of H_2_O_2_ into high toxicity ·OH via a Fenton-like reaction, which has a good CDT effect [28,29]. Norcantharidin (NCTD), a classic small molecule anticancer drug used in cancer CT, is a demethylated analogue of the natural toxic compound cantharidin (CTD) isolated from Chinese traditional medicine blister beetle (Mylabris) [30,31]. NCTD is known to be an inhibitor of serine/threonine protein phosphatase 2A (PP2A) [32], which has a certain ability to damage the DNA of tumor cells. At present, many NCTD preparations have been used in the treatment of primary liver cancer in China [33,34]. At the same time, numerous studies have investigated the mechanism of action of NCTD on colon cancer cell lines (such as CT26 and HT29) [35,36], as well as breast cancer cells (MCF-7) [37]. However, the short biological half-life, poor water solubility and high toxicity of NCTD limit its application in clinical therapy [[38], [39], [40]]. In addition, PEG is a polymer with good water solubility and compatibility with many organic components, and PEGylation can increase the stability of blood circulation by protecting itself from clearance by the reticuloendothelial system [41,42]. Thus, the connection of NCTD and NH_2_-PEG-NH_2_ (hereinafter referred to as PEG) via acid-sensitive amide bonds can both increase the time of the NCTD in the blood circulation and promote the entry of the target drug into the tumor cell on a weak acidic switch [43,44].

**Scheme 1:**
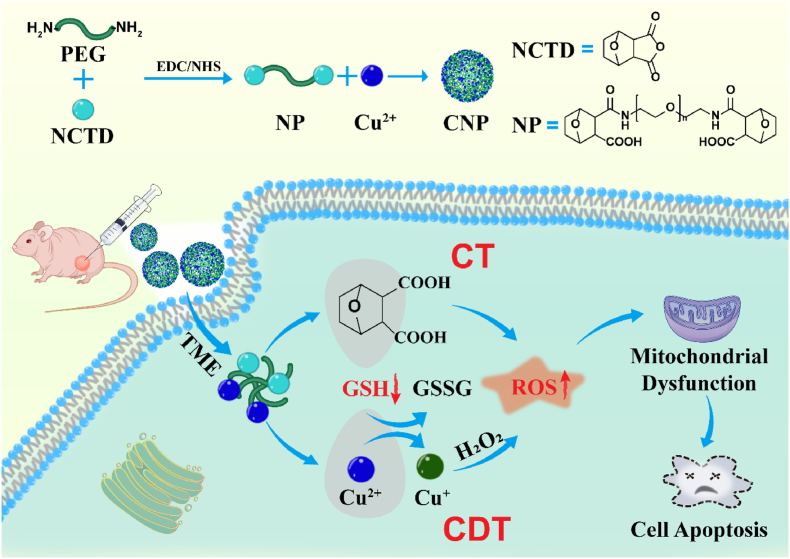
Schematic illustration of the synthesis of Norcantharidin/Cu^2+^ dual-depleting GSH and pH-responsive nanocatalyst for CT/CDT synergistic tumor therapy.

Herein, we developed a NCTD/Cu^2+^ dual-depleting GSH and pH-responsive nanocatalyst integrating CT and CDT, and investigated the therapeutic efficacy of the nanocatalyst in combination with CT/CDT for breast cancer treatment (as shown in Scheme 1). NCTD and PEG were first connected through an amide reaction, hoping to improve the short half-life of NCTD and increase the time of the catalyst in the blood circulation. Then Cu^2+^ and NCTD-PEG (NP) were coordinated to form a nanocatalyst Cu-NCTD-PEG (CNP). When reached the tumor, due to the weak acidic of TME, the catalyst released NCTD and Cu^2+^ in pH response, and both consumed GSH and generated ROS, which reduced the antioxidant capacity of cancer cells while increasing higher oxidative stress, thus destroying cell REDOX homeostasis and leading to mitochondrial dysfunction. Finally, apoptosis of tumor cells was induced to achieve high-efficiency CT and CDT synergistic tumor therapy.

## 2 Experimental section

### 2.1 Materials

NCTD and 5,5-dimethyl-1-pyrroline-N-oxide (DMPO) was obtained from Bide Pharmatech Co. Ltd. NH_2_-PEG-NH_2_, CuCl_2_, NHS, EDC·HCl, and NaOH were obtained from Aladdin Biochemical Technology Co. Ltd. 5,5′-dithiobide (2-nitrobenzoic acid) (DTNB) and 3-(4,5-dimethylthiazole-2-yl)-2,5-diphenyl tetrazolium bromide (MTT) were sourced from Solarbio Science & Technology Co. Ltd. Reduced GSH Content Assay Kit purchased from Sangon Biotech Co. Ltd. Mito-Tracker Red CMXRos, Mitochondrial Membrane Potential Assay Kit with JC-1, Enhanced ATP Assay Kit, ROS Assay Kit, Singlet Oxygen Sensor Green Fluorescent Probe, Reactive Oxygen Species Assay Kit for Superoxide Anion with DHE and superoxide dismutase (SOD) were purchased from Beyotime Biotechnology Co. Ltd. Hydroxyl Radical Detection Kit was purchased from Bestbio Biotechnology Co. Ltd. Other chemical reagents were obtained from Xilong Scientific Co. Ltd. Tumor cell lines and normal cell lines were sourced from the cell bank of the Chinese Academy of Sciences. The study utilized 6-8 weeks-old female BALB/c mice and 5-6 weeks-old female SPF BALB/c nude mice, which were acquired from Ziyuan Laboratory Animal (Hangzhou) Technology Co. Ltd.

### 2.2 Preparation of NCTD-PEG (NP)

NP is a slightly modified synthesis based on the method described in the literature [45]. 100 mg PEG (0.05 mmol) with different molecular weights (2k, 4k or 6k) was selected, 23 mg NHS (0.20 mmol), and 31 mg EDC (0.20 mmol) were dissolved in water, the mixture was stirred in the dark for 4 h at room temperature, followed by the addition of 34 mg of NCTD (0.20 mmol) and further stirring for an additional 24 h. Following the freeze-drying process, the product was washed with acetone and methylene chloride, and subsequently dried at 60 °C, resulting in a yellow oil.

### 2.3 Preparation of Cu-NCTD-PEG (CNP) and Cu-PEG (CP)

40 mg NP (0.02 mmol) and 24 mg NaOH (0.6 mmol) were dissolved in water and added to an aqueous solution of 40.5 mg CuCl_2_ (0.3 mmol). Then stirred for 30 min at room temperature, and centrifuged to discard the supernatant. It was subsequently washed twice with ethanol and water, then dried the samples in an oven at 60 °C. The control CP follows the same synthesis procedure, with the exception that NCTD-PEG is replaced with PEG.

### 2.4 Characterization

The catalyst's morphology was analyzed using transmission electron microscopy (TEM) (Talos F200). The chemical compositions and structures of the samples were studied using an X-ray diffractometer (XRD) (Rigaku DMAX2500). The zeta potential and particle size were analyzed using a nano-size and zeta potential analyzer (Zetasizer Nano ZS). The UV–visible absorption spectrum was recorded using an ultraviolet–visible spectrophotometer (UV-2600). The functional groups of the catalyst were identified using Fourier transform infrared spectroscopy (FT-IR) (Spectrum Two). The chemical composition and structure of samples were analyzed using X-ray photoelectron spectroscopy (XPS) (Nexsa). The metal content of the catalyst was determined using inductively coupled plasma mass spectrometry (ICP-MS) (NexION 300X). The formation of ·OH was detected using electron paramagnetic resonance spectroscopy (EPR) (A300). Cell viability, intracellular GSH content, and adenosine triphosphate (ATP) content were assessed using a multifunctional cell imaging system (Cytation5). Cell morphology was observed using an automated cell imaging system (EVOS FL Auto 2). Apoptosis was evaluated using flow cytometry (FACS Verse). The CNP nanomaterials were dispersed in various physiological environments (PBS, RPMI 1640, RPMI 1640 + serum, RPMI DMEM, RPMI DMEM + serum), and subsequently incubated at controlled temperatures for varying time periods to systematically evaluate their dispersion stability and sedimentation behavior.

### 2.5 Release of metal ion

The process of copper release from CNP was monitored by dialysis method. Briefly, 1 mL of CNP was placed in a dialysis bag (MWCO 1000 kDa) and dialyzed under various conditions. At specified time intervals, samples of the dialysate were collected and replaced with fresh PBS. After dilution, the copper content in the samples was quantified by ICP-MS.

### 2.6 GSH and H_2_O_2_ consumption

In a PBS solution containing 50 μg/mL NCTD, CP, or CNP, 0.2 mM GSH was added and incubated at 37 °C for varying time periods. Subsequently, 0.025 μg/mL DTNB was added and incubated for 5 min. The change of absorbance at 412 nm was then measured by a UV–vis spectrometer. NCTD, CP, CNP_2k_, CNP_4k_, and CNP_6k_ (50 μg/mL) were incubated with GSH (0.2 mM) in PBS solution at 37 °C for different periods of time. The generation and consumption of H_2_O_2_ were detected according to the hydrogen peroxide detection kit. All subsequent experiments used CNP_2k_ unless otherwise specified.

### 2.7 ROS detection

In a solution comprising 10 μg/mL of methylene blue (MB), 0.3 % H_2_O_2_, 50 μg/mL NCTD, CP, or CNP, and 0.2 mM GSH, the sample was mixed and incubated for 30 min at 37 °C. To assess the generation of ·OH through the Cu^2+^/Cu^+^-mediated Fenton-like reaction and their scavenging by GSH, the change in absorbance at 665 nm was measured using a UV–vis spectrometer. The impact of varying concentrations of GSH (0, 0.1, 0.2, 0.3, 0.4, and 0.5 mM) was also evaluated and CNP (0, 5, 10, 25, 50, and 100 μg/mL) on MB degradation were examined under these experimental conditions. In a solution comprising 0.4 mM GSH,100 μg/mL NCTD, CP, or CNP, and 0.6 % H_2_O_2_ (the concentration is 0.2 M), the mixture was incubated for 30 min at 37 °C. Subsequently, 1 μL of DMPO was added, followed by an additional incubation period of 5 min. The signal peak was detected by EPR. In addition, detected the generation of ·OH, ^1^O_2_, and O_2_^•−^ at different time points using TMB, DPBF, and DHR123, respectively. Specifically, TMB (200 μM), DPBF (20 μg/mL), DHR123 (1 mM), H_2_O_2_ (0.3 %), and GSH (0.2 mM) were added either individually or in various combinations. The detection was subsequently performed using UV–vis spectroscopy and fluorescence spectroscopy.

### 2.8 In vitro cell activity and toxicity

The cytotoxicity and cell viability associated with the catalyst were evaluated using the standard MTT assay. Cell suspensions were inoculated in 96-well plates and cultured in a sterile environment at 37 °C. When the cell confluence reached 60–70 % confluence, the drug was added at concentrations of 0, 1.56, 3.12, 6.25, 12.5, and 25 μg/mL. Following 24 h incubation, 10 μg/mL of MTT was added to the plates, which were then incubated for an additional 4–6 h. Afterward, the liquid was discarded, and 100 μL of DMSO was added to each well. The plates were shaken for 10 min to ensure complete dissolution of the purple formazan crystals. Finally, the absorbance at 570 nm was measured for each well (n = 5) using a multifunctional cell imaging system.

### 2.9 Cell uptake

Cell suspensions were inoculated in 7 cm petri dishes and cultured in the sterile environment at 37 °C. When the cells reached a confluence of 60–70 % confluence, CNP or PBS was added. After incubation for 24 h, wash the cells multiple times with PBS, and the cells were treated and transferred to a centrifuge tube. They were then centrifuged at 1000 rpm for 10 min, after which the supernatant was discarded. Subsequently, the cells were washed twice with PBS by centrifugation. After removing PBS, 1 mL of nitric acid and 500 μL of 30 % H_2_O_2_ were added. The mixture was incubated 24 h. After appropriate dilution of the sample, the copper content was determined by ICP-MS.

### 2.10 GSH level

Cell suspensions were inoculated in 7 cm petri dishes and cultured in the sterile environment at 37 °C. When the cells reached a confluence of 60–70 % confluence, NCTD, CP, CNP or PBS was added. After incubation for 24 h, the cells were treated and transferred to a centrifuge tube. They were then centrifuged at 1000 rpm for 10 min, after which the supernatant was discarded. Subsequently, the cells were washed twice with PBS by centrifugation. Next, the suspended cells were processed following the protocol provided in the GSH assay kit instructions, and the cells were repeatedly frozen and thawed 3 times. The cells were centrifuged at 8000 g and 4 °C for 10 min, and collected the supernatant. The absorbance at 412 nm was measured using a multifunctional cell imaging system.

### 2.11 Cell apoptosis

Cell suspensions were inoculated in 6-well plates and cultured for 24 h in the sterile environment at 37 °C. When the cells reached a confluence of 60–70 % confluence, NCTD, CP, CNP or PBS was added. After incubation for 24 h, the cells were treated and transferred to a centrifuge tube. They were then centrifuged at 1000 rpm for 10 min, after which the supernatant was discarded. Subsequently, the cells were washed twice with PBS by centrifugation. The cells were collected, washed, and stained according to the Annexin V-FITC/PI apoptosis kit instructions and analyzed by flow cytometry.

### 2.12 Calreticulin (CRT) immunofluorescence detection

Following the seeding of the plate and subsequent treatment with the drug for 8 h, the plate was subjected to multiple washes with PBS. Cells were then fixed using a cell fixation solution for 20 min, followed by additional washes with PBS. The cells were permeabilized with 0.5 % Triton X-100 for 20 min, washed again with PBS, and blocked with 3 % BSA-PBS at room temperature for 30 min. After discarding the blocking solution, the primary antibody (Rabbit mAb) was added and incubated overnight at 4 °C. On the following day, the plate was incubated at room temperature for 30 min, washed with 0.2 % PBST, and subsequently incubated with the diluted fluorescent secondary antibody (Goat Anti-Rabbit IgG H&L) in the dark for 1 h. Following further washes with PBST, DAPI was added and incubated in the dark for 10 min. Finally, after washing with PBST, the plate was examined under a confocal microscope.

### 2.13 Mitochondrial fluorescence staining

Cell suspensions were inoculated in 6-well plates and cultured in the sterile environment at 37 °C. When the cells reached a confluence of 60–70 % confluence, NCTD, CP, CNP or PBS was added. The medium was removed after incubation for 12 h. Subsequently, the cells were washed twice with PBS by centrifugation. Then, the cells were stained with mito-tracker red dye for 20 min. Finally, the mitochondrial red fluorescence signal was observed using the cell imaging system.

### 2.14 Mitochondrial membrane potential (MMP)

Cell suspensions were inoculated in 6-well plates and cultured in the sterile environment at 37 °C. When the cells reached a confluence of 60–70 % confluence, the cells were treated with RPMI 1640 medium supplemented with 10 % (v/v) FBS, along with 12.5 μg/mL of NCTD, CP, or CNP, and incubated for 12 h. Following incubation, the medium was aspirated, and the cells were gently washed twice with PBS. Subsequently, the cells were stained in accordance with the JC-1 kit protocol, and intracellular fluorescence intensity was quantified using an automated cell imaging system.

### 2.15 ATP level

Cell suspensions were inoculated in 6-well plates and cultured in the sterile environment at 37 °C. When the cells reached a confluence of 60–70 % confluence, NCTD, CP, CNP or PBS was added. After incubation for 24 h, the cells were lysed and treatment according to the instructions of the enhanced ATP detection kit and then transferred to a light-tight 96-well plate for detection using a multifunctional cell imaging system.

### 2.16 Inhibitory effect of SOD and catalyst co-incubation on cell activity

The inhibitory effect of co-incubation of SOD and the catalyst on cell activity was determined using the standard MTT method. Cell suspensions were seeded in 96-well plates and incubated in sterile conditions at 37 °C. Once the cells reached 60–70 % confluence, except for the control group, NCTD, CP, or CNP were added for 24 h. Alternatively, 900 U/mL SOD was added for 1 h, that was followed by adding NCTD, CP, or CNP and incubation for 24 h. Next, 10 μg/mL MTT was added to the wells and incubated for 4–6 h. Following this, the medium was discarded, and 100 μL of DMSO was added to each well to dissolve the formazan crystals. The plate was then placed on a shaker for 10 min to ensure complete dissolution of the purple formazan crystals. Absorbance at 570 nm was measured for each well (n = 5) using a multifunctional microplate reader.

### 2.17 Flow cytometry for intracellular ROS detection

Cell suspensions were inoculated in 6-well plates and cultured in the sterile environment at 37 °C. When the cells reached a confluence of 70 % confluence, RPMI 1640 medium (containing 10 % FBS), 12.5 μg/mL NCTD, CP and CNP were added. After incubation for 8 h, the cells were digested with trypsin, and the cell suspension was collected and transferred to a centrifuge tube. The suspension was centrifuged at 1000 rpm for 10 min, the supernatant was removed, and the cells were washed twice with PBS by centrifugation. Then, the cells were stained according to the instructions of the ROS detection kit and passed through the membrane, and detected by flow cytometry.

### 2.18 ROS staining

The cell suspension was seeded in 6-well plates and incubated under sterile conditions at 37 °C. Once the cells reached 60–70 % confluence, except for the control group, NCTD, CP, or CNP were added for 8 h. Alternatively, 900 U/mL SOD was added for 1 h, that was followed by adding NCTD, CP, or CNP and incubation for 8 h. The supernatant was carefully discarded, and the cells were washed twice with PBS. In accordance with the ROS kit protocol, the cells were stained, and the intracellular fluorescence intensity was quantified using an automated cell imaging system.

### 2.19 ROS species

The cell suspension was seeded in 6-well plates and incubated understerile conditions at 37 °C. When the cells reached 60–70 % confluence, RPMI 1640 medium (containing 10 % FBS), 12.5 μg/mL NCTD, CP or CNP was added for incubation for 8 h. The medium was removed and the cells were gently washed twice with PBS. The cells were stained with the corresponding dyes and the intracellular fluorescence intensity was observed using an automatic cell imaging system.

### 2.20 In vivo therapy

All animal experiments were conducted in Animal Laboratory Central, Guangxi Normal University after approval by the Experimental Animal Ethics Committee of Guangxi Normal University (ref: GXNU-202403-043). Before conducting *in vivo* therapeutic experiments, we delivered fluorescently labeled CNP nanoparticles into animals via in situ injection, and monitored their distribution in real time using *in vivo* imaging technology. Healthy BALB/c mice were used to collect whole blood from the eyeball and immediately diluted with Saline. Control group and experimental groups with H_2_O (positive control), 0.9 % Saline (negative control), and with different concentrations of CNP were set up. After centrifugation at 3000 rpm for 20 min, pictures were taken. The therapeutic effects of NCTD, CP, and CNP *in vivo* were studied in a 4T1 tumor-bearing mouse model. Briefly, When the tumor volume reached approximately 60–100 mm^3^, the mice were randomly assigned to four treatment groups: Saline, NCTD, CP, and CNP, with five animals in each group. Mices were administered in situ (20 μL, 2 mg/mL) every 2 days, and body weight and tumor volumes were measured every 2 days. In addition, an evaluation of lung metastasis suppression was performed. BALB/c mice aged 6–8 weeks were injected with 4T1 cells (1 × 10^6^) via the tail vein. 5 days later, Saline or CNP (100 μg/mL, 100 μL) were administered via the tail vein every other day. 14 days later, the lungs from each group were carefully excised, photographed, and further analyzed.

## 3 Results and discussion

### 3.1 Synthesis and characterization

**Fig. 1:**
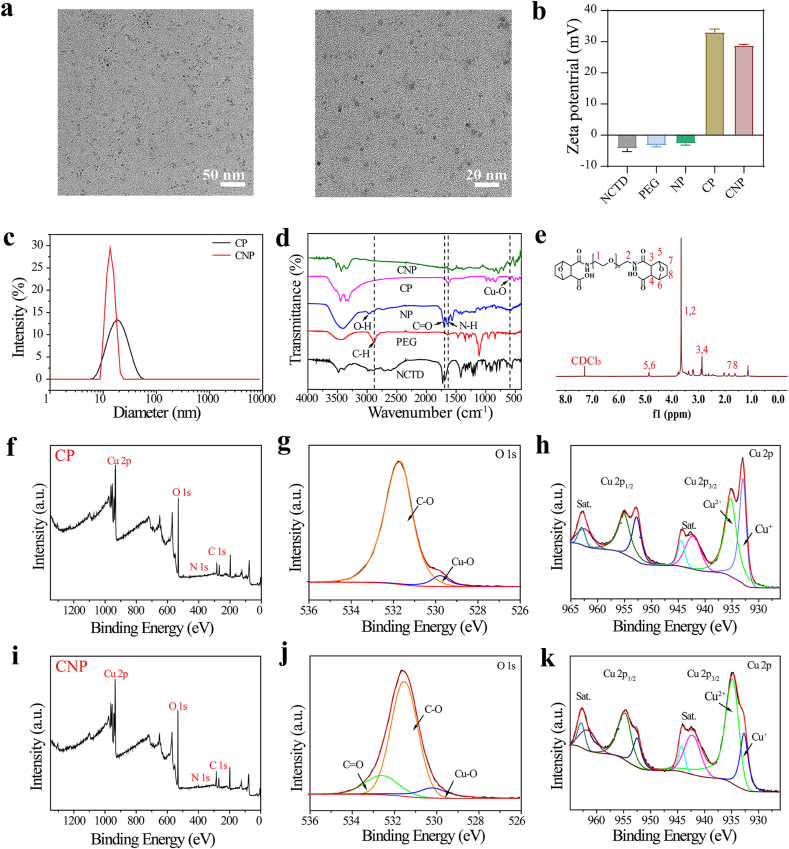
(a) TEM of CNP at different magnifications. (b) Zeta potentials of NCTD, PEG, NP, CP, and CNP. (c) Hydration particle size of CP and CNP. (d) FT-IR spectra of NCTD, PEG, NP, CP, and CNP. (e) ^1^H NMR spectrum of NP in CDCl_3_. (f–h) XPS spectra of CP. (i–k) XPS spectra of CNP.

**Fig. 2:**
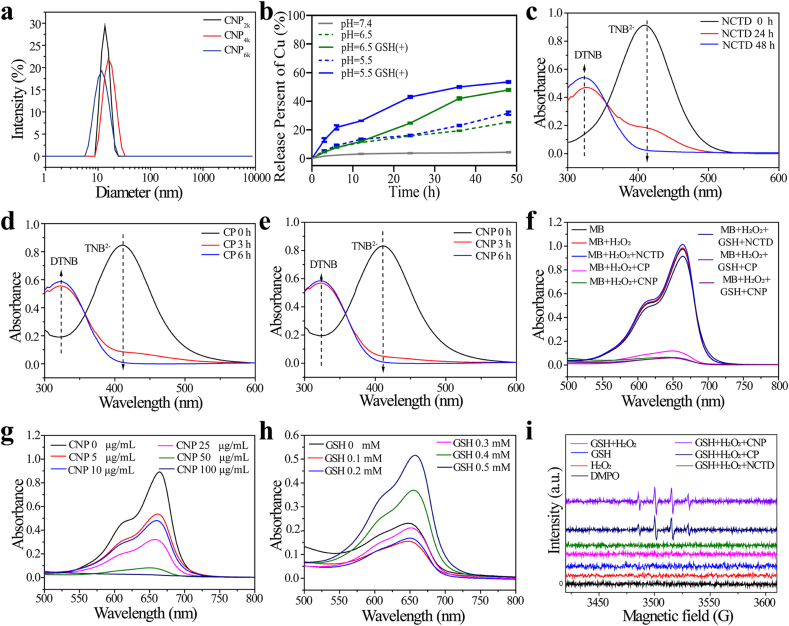
(a) Size of CNP_xk_ synthesized with PEG of different chain lengths (CNP elsewhere in the paper are CNP_2k_ unless otherwise noted). (b) The release of copper over time with different pH and presence of GSH. (c–e) DTNB experiments of NCTD, CP, and CNP. Degradation ability of MB under different conditions: (f) add different substrates; (g) different concentrations of CNP; (h) different GSH concentrations. (i) EPR spectra of NCTD, CP and CNP under different conditions.

NCTD and PEG were first linked through an amide reaction, and Cu^2+^ and NP were then coordinated through Cu-O to form a nanocatalyst (Scheme 1). The TEM image (Fig. 1a) revealed that CNP was in the shape of nanodots. High-resolution TEM (HRTEM) reveals a discernible lattice structure (Fig. S1). However, from the XRD test results, the XRD pattern of CNP does not have sharp diffraction peaks (Fig. S2), so it may be a mixed structure of crystalline and amorphous states. The zeta potential (Fig. 1b) and hydrodynamic particle size of CNP (Fig. 1c) were analyzed using a nano size and zeta potential analyzer. The size of CNP was 14.27 ± 1.05 nm, while the size of CP, used as a control, was 16.79 ± 0.07 nm, slightly larger than CNP. The zeta potentials of CP and CNP were 33.10 ± 0.96 mV and 28.83 ± 0.35 mV, respectively. The UV–vis spectrum (Fig. S3) showed a redshift in the absorption peak wavelength after the coordination of NP with Cu^2+^. FT-IR spectroscopy (Fig. 1d) indicated that the vibration peak near 1628 cm^−1^ is attributed to N-H, while the new C=O and -OH stretching vibration absorption peaks of NP appeared near 1702 cm^−1^ and 2975 cm^−1^, respectively, due to the ring-opening of PEG after reaction with NCTD. This suggests the successful synthesis of NP. Additionally, the absorption peak around 587 cm^−1^ was attributed to Cu-O, indicating the successful coordination of Cu^2+^ and NP. The ^1^H NMR spectra (Fig. 1e) showed that compared with NCTD (Fig. S4), a new hydrogen characteristic peak (3.20–3.76 ppm) appeared in the ^1^H NMR spectra of NP, attributed to the PEG in conjugation, confirming the successful synthesis of NP. The surface element composition and metal valence state of CNP were further investigated using XPS. XPS spectra of CP (Fig. 1f–h) and CNP (Fig. 1i–k) both contained C, O, N, and Cu elements (the proportion of Cu elements in CNP, as detected by ICP-MS was 39.6 %). Fitting the O 1s of CNP (Fig. 1j) revealed binding energies of 532.58 eV, 531.70 eV, and 530.18 eV, attributed to C=O, C-O, and Cu-O, respectively. The increased binding energy of C=O compared with CP further demonstrated the successful connection of NCTD. Similarly, fitting the Cu 2p (Fig. 1h and k) showed binding energies near 932.7 and 934.8 eV, attributed to Cu^+^ and Cu^2+^, respectively. The presence of Cu ^+^ indicated that the Cu^2+^ from the raw material CuCl_2_ was reduced to Cu^+^. Copper in low valence state can enhance the efficiency of the Fenton-like reaction, suggesting that both CP and CNP have good ability to produce ROS. These characterizations confirmed the successful synthesis of CNP. Based on this, CNP_xk_ was synthesized by varying the PEG chain length (Mw = 2k, 4k, 6k) to investigate the effect of PEG chain length on catalyst size. The particle size of CNP_xk_ was measured using a nano-size and zeta potential analyzer. As shown in Fig. 2a, the particle size of CNP_xk_ were similar, which indicated changing the PEG chain length had little effect on the catalyst size. In addition, the dispersibility and stability of CNP in physiological environments were systematically evaluated. CNP was dispersed in various media, including PBS, RPMI 1640, RPMI 1640 + serum, RPMI DMEM, and RPMI DMEM + serum, and monitored over a period of seven days through photographic documentation. As shown in Fig. S5, no significant sedimentation was observed on the seventh day, suggesting that CNP exhibits excellent stability and is highly suitable for further biological application research in physiological environments.

### 3.2 Chemodynamic properties

Firstly, we simulated different physiological conditions outside the cell to detect the copper release capacity using ICP-MS. As shown in Fig. 2b, under the condition of neutral pH 7.4, the copper release was minimal, and it gradually increased with decreasing pH. In the simulated TME, at pH 5.5 and in the presence of GSH, the copper release was the highest, indicating that CNP is acid-sensitive and can achieve controlled release. Next, we qualitatively tested whether NCTD, CP, and CNP can react with GSH. After reacting with GSH for a certain period, the UV–vis spectra (Fig. S6) changed, showing that all three compounds can consume GSH. Additionally, the reaction of DTNB with GSH produces yellow 5-thio-2-nitrobenzoic acid, which has a maximum absorption peak at 412 nm. Therefore, we evaluated the GSH consumption capacity of NCTD, CP, and CNP by studying the changes in absorbance values at 412 nm after different reaction times with GSH. As shown in Fig. 2c–e, all three compounds can cause DTNB to fade, but NCTD took the longest time, followed by CP, and CNP consumed GSH most efficiently, causing the highest rate of DTNB fading. By investigating the impact of varying PEG molecular weights on GSH consumption, it was observed that the CNP_2k_ resulted in significantly greater GSH consumption compared to CNP_4k_ and CNP_6k_ (Fig. 2e and S7). This confirms that CNP can release Cu^2+^ under acidic conditions, and a Fenton-like reaction occurs after Cu^2+^ consumes GSH. Through the investigation on the effects of CNP with different PEG molecular weights on the generation and consumption of H_2_O_2_, it was found that CNP_2k_ was significantly superior to CNP_4k_ and CNP_6k_ in promoting the generation of H_2_O_2_, while in consuming H_2_O_2_, it was basically equivalent to CNP_4k_ and CNP_6k_ (Fig. S8). Additionally, we investigated the effects of different substrates on MB degradation (Fig. 2f and S9a). After adding H_2_O_2_ and CNP to MB, regardless of whether GSH was added, the MB degradation effect was significant, proving that CNP can produce highly toxic ROS. When GSH was not added, Cu^+^ reacted with H_2_O_2_ to generate the partial highly toxic ·OH. When GSH was added, Cu^2+^ consumed GSH and produced a Fenton-like reaction, enhancing the CDT effect. Next, we investigated the effects of different CNP concentrations on MB degradation (Fig. 2g and S9b). As the CNP concentration increased, the intensity of the absorption peak at 665 nm progressively diminished, suggesting that the generation of ROS (·OH, ^1^O_2_, and O_2_^•−^) is concentration-dependent on CNP. We then examined the impact of varying GSH concentrations on MB degradation in the presence of H_2_O_2_ and CNP (Fig. 2h and S9c). In the range of 0–0.1 mM, as the GSH concentration increased, the absorption peak intensity at 665 nm progressively diminished as Cu^2+^ reacted with GSH to form Cu^+^ and GSSG, which was followed by the reaction of Cu^+^ with H_2_O_2_ to generate ·OH radicals, ultimately resulting in MB degradation. However, when the GSH concentration surpassed 0.2 mM, the absorption peak at 665 nm intensified due to the excessive GSH scavenging the produced ROS. The MB degradation experiment revealed that CNP exhibits a robust capacity for generating ROS.

**Fig. 3:**
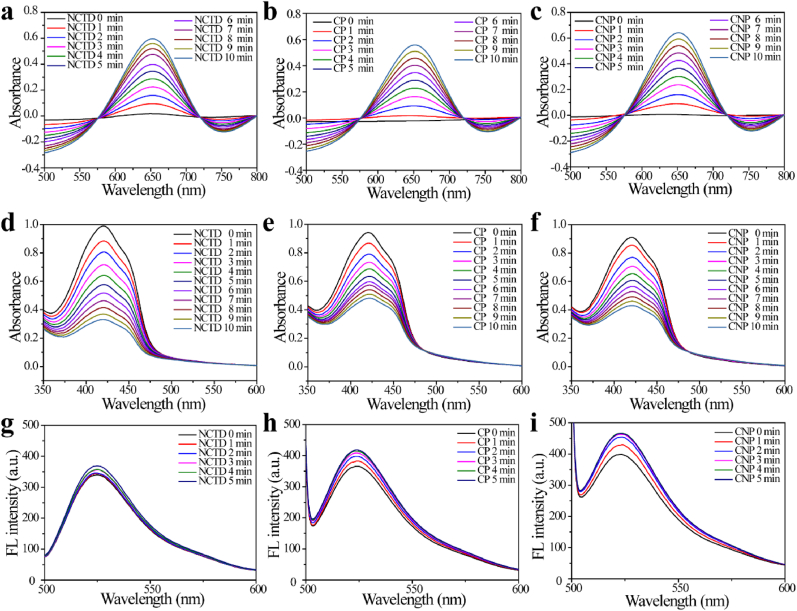
The generation of ·OH when TMB was incubated with (a) NCTD + H_2_O_2_, (b) CP + GSH + H_2_O_2_, and (c) CNP + GSH + H_2_O_2_ for different periods of time; the generation of ^1^O_2_ when DPBF was incubated with (d) NCTD + H_2_O_2_, (e) CP + H_2_O_2_, and (f) CNP + H_2_O_2_ for different periods of time; the generation of O_2_^•−^ when DHR123 was incubated with (g) NCTD + H_2_O_2_, (h) CP + H_2_O_2_, and (i) CNP + H_2_O_2_ for different periods of time.

To further verify the generation of ROS, we conducted EPR spectroscopy. According to the EPR spectra (Fig. 2i), CNP reacting with GSH and H_2_O_2_ would generate a 1:2:2:1 EPR signal peak, which is characteristic of the ·OH radical. EPR spectrum in Fig. 2i does not show the corresponding signal peak for O_2_^•−^, This is because GSH has a strong antioxidant capacity, and O_2_^•−^ is unstable in nature and is easily converted by GSH into relatively stable H_2_O_2_. Subsequently, the production capabilities of ·OH, ^1^O_2_ and O_2_^•−^ by CNP were quantitatively assessed using TMB, DPBF, and DHR123, respectively. Compared with the control group (Fig. S10), the results demonstrated that CNP can enhance the generation of ·OH, ^1^O_2_, and O_2_^•−^ to a certain extent (Fig. 3), thereby exhibiting promising CDT efficacy.

### 3.3 Anti-tumor effect in vitro

**Fig. 4:**
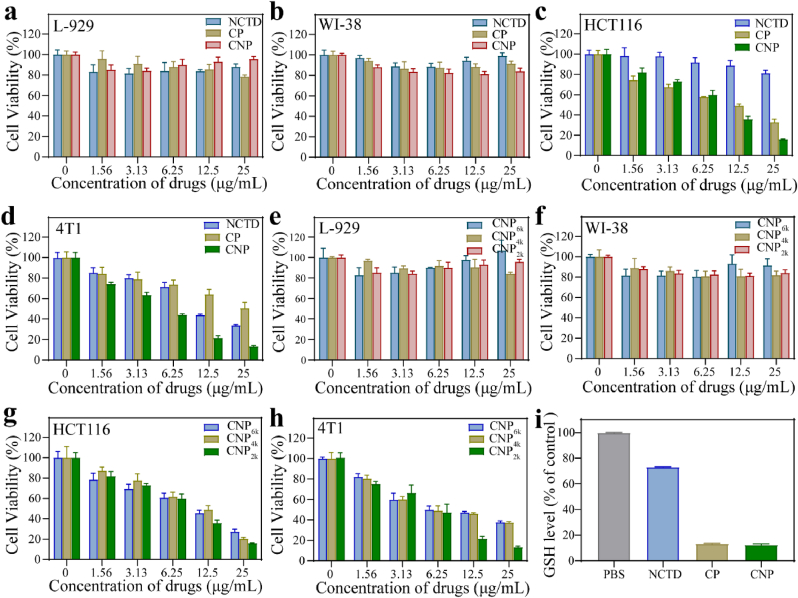
Relative cell viability of (a) L-929, (b) WI-38, (c) HCT116, and (d) 4T1 cells after incubation with different concentrations of NCTD, CP, or CNP. Relative cell viability of (e) L-929, (f) WI-38, (g) HCT116, and (h) 4T1 cells after incubation with CNP_xk_. (i) GSH level with different treatments of 4T1 cells.

In view of the excellent ·OH production ability of CNP, we further investigated its cytotoxicity against L-929 and WI-38 cells and its antitumor activity against HCT116 and 4T1 cells using the MTT assay. The normal cells, including L-929 (Fig. 4a) and WI-38 (Fig. 4b) cells maintained a high relative cell activity after 24 h treatment with 25 μg/mL CNP, indicating that the nanocatalyst had good stability under neutral conditions and did not decompose prematurely, thus exhibiting low toxicity to normal cells. In contrast, after treatment with different concentrations of CNP for the same duration, the cell survival rates of HCT116 (Fig. 4c) and 4T1 (Fig. 4d) cells decreased gradually with increasing concentration. At the highest concentration, the cell survival rates were only 16.01 % and 13.15 %, respectively, indicating that CNP had high antitumor activity. Similarly, we found that the synergistic effect of CNP was better than that of NCTD with CT alone and CP with CDT alone. Next, we also explored the cytotoxicity of CNP_xk_. As shown in Fig. 4e–h, CNP_xk_ exhibited similar cytotoxicity and cell activity, but CNP_2k_ had the best effect.

The amount of metal taken up by individual cells was detected. As shown in Fig. S11, the results indicated that tumor cells exhibited better cellular uptake of CNP than normal cells. After metal uptake by cells, a Fenton-like reaction occurred because of the high level of GSH within cancer cells. Therefore, we examined the changes of GSH levels after 24 h of interaction between catalysts and 4T1 cells. As shown in Fig. 4i and S12, compared with the control, the GSH levels in the treatments of NCTD, CP, and CNP groups decreased to 73.08 %, 13.34 %, and 12.40 %, respectively. NCTD and CP both indicate ability to reduce GSH, and CNP dually consumed GSH, which was consistent with the above trend of extracellular GSH consumption. But compared with the CP group, there is no significant decrease (less than 1 %) in the CNP group. This is primarily due to that as GSH is consumed, its concentration decreases substantially, making it more challenging to further deplete GSH under low-concentration conditions.

**Fig. 5:**
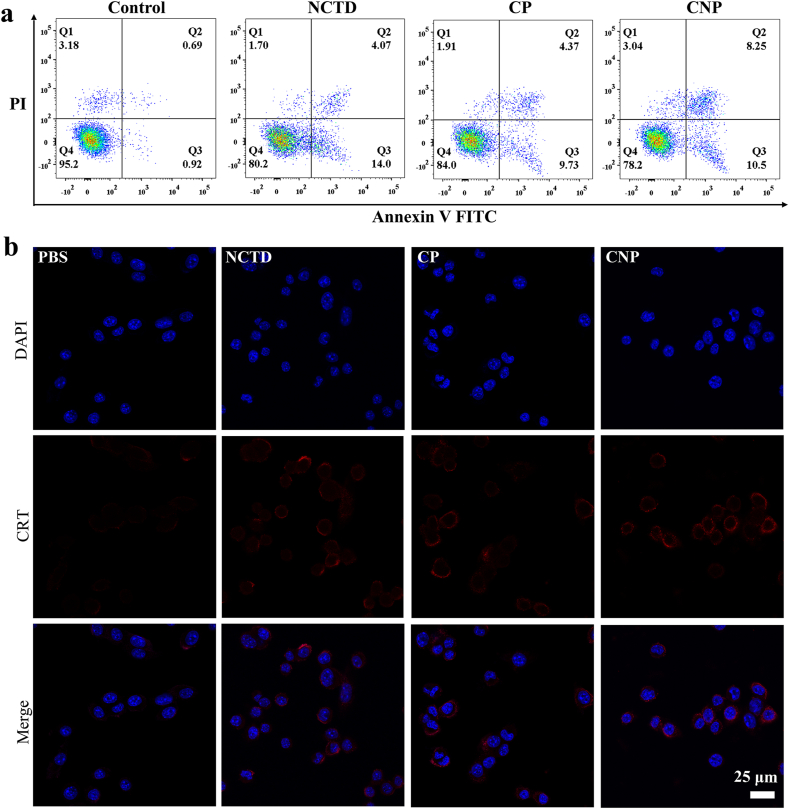
(a) Cell apoptosis effects with different treatments of 4T1 cells. (b) Fluorescence imaging of CRT in 4T1 cells after 8 h incubation with PBS, NCTD, CP and CNP.

Furthermore, we explored how the nanocatalyst induces cell death. The effect of the nanocatalyst on apoptosis was measured by flow cytometry. As shown in Fig. 5a, compared to the control, the percentage of apoptotic cells in the drug-treated groups increased significantly compared to the control group. Specifically, after treatment with the catalyst on 4T1 cells, the percentage of apoptotic cells (including early Q3 and late Q2 apoptosis rates) increased from 18.07 % for NCTD with CT alone, 14.10 % for CP with CDT alone, to 18.75 % for CNP acting synergistically. Clearly, the synergistic effect of CNP was significantly stronger than that of NCTD and CP in inducing apoptosis of 4T1 cells, which was consistent with the MTT activity trend. CRT is a calcium-binding chaperone protein primarily localized in the lumen of the endoplasmic reticulum (ER). During the early stages of apoptosis, following the depletion of ER Ca^2+^ stores and the modification of endoplasmic reticulum membrane permeability, CRT translocates from the ER lumen to the cell surface. Consequently, we initially investigated the expression of CRT under the influence of various materials using immunofluorescence analysis. As shown in Fig. 5b, CRT is translocated from the cytoplasm to the cell membrane, where it becomes exposed. Notably, the response of CRT on the cell membrane is significantly enhanced following treatment with CNP.

**Fig. 6:**
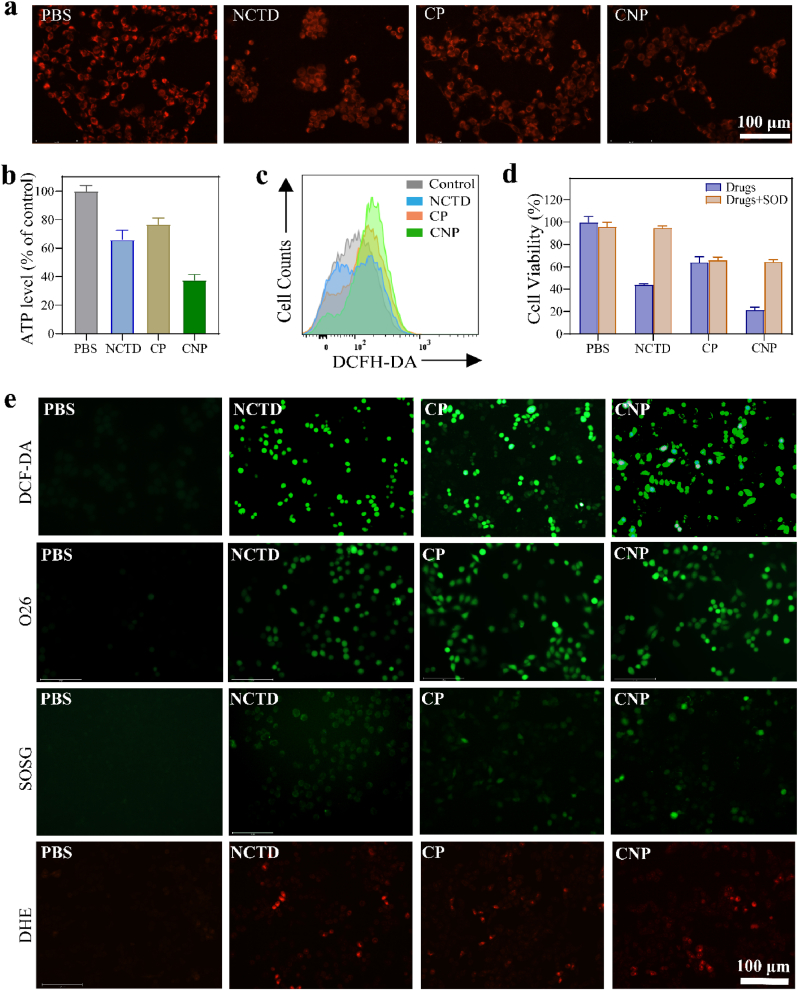
The effects of different treatments of 4T1 cells: (a) Mitochondrial red fluorescence staining (red fluorescence), (b) ATP level, (c) ROS levels were detected using flow cytometry, (d) Relative cell activity of 4T1 cells incubated with SOD (900 U/mL) and catalyst. (e) ROS fluorescence staining, detected the intracellular generation of ·OH, ^1^O_2_ and O_2_^•−^ using the fluorescent probes O26, SOSG, and DHE, respectively. (For interpretation of the references to colour in this figure legend, the reader is referred to the Web version of this article.)

Since some apoptosis induced by antitumor drugs is mediated by ROS production and mitochondrial pathways [20,46], we further investigated the apoptosis pathways of 4T1 cells after confirming that the nanocatalyst could induce apoptosis. Mitochondria are the energy source of cells, supplying energy in the form of ATP and affecting the basic functions of many cells. Damage to mitochondria leads to dysfunction involving many pathological processes [47,48]. First, after mitochondrial staining of 4T1 cells treated with different treatments (Fig. 6a), it was found that the cell density and red fluorescence intensity decreased in the treatment of CNP, indicating mitochondrial damage. After mitochondrial damage, the MMP of 4T1 cells was assessed using the JC-1 fluorescent probe. As shown in Fig. S13, following CNP treatment, the red fluorescence indicative of high MMP progressively shifted to green fluorescence associated with low MMP, indicating that CNP could dissipate the MMP of cells. Subsequently, we further examined whether the catalysts could affect ATP production in the cells. Have conducted multiple sets of parallel experiments, after treatment with NCTD, CP, or CNP, the results showed that CNP reduced the ATP level of the cells the most, to 37.63 % (Fig. 6b and S14) [49]. These data indicate that CNP can effectively inhibit ATP levels in cells, disrupt mitochondrial function, and ultimately lead to mitochondrial dysfunction. Subsequently, the intracellular ROS levels were detected using flow cytometry. As shown in Fig. 6c, after CNP acted on 4T1 cells, compared with the Control group, there was a significant rightward shift, indicating an increase in ROS content.

Additionally, several researches have shown that damaged mitochondria are related with changes in intracellular ROS levels [[50], [51], [52]]. Therefore, we investigated intracellular ROS levels using the MTT assay and ROS fluorescence probe. SOD is an enzyme that can catalyze the disproportionation of O_2_^•−^ into O_2_ and H_2_O_2_. Therefore, we first studied the effect of co-incubation of SOD and the catalyst on cell activity. As shown in Fig. 6d, compared with the treatment of NCTD, the relative cell activity of the treatment of NCTD + SOD was similar with the control (including PBS and PBS + SOD), indicating that the toxic O_2_^•−^ produced by NCTD after cell treatment was catalyzed by SOD into non-toxic H_2_O_2_ and O_2_, thereby reducing drug activity and increasing cell survival rate. This suggests that O_2_^•−^ produced by NCTD has a significant effect on the activity of tumor cells. Similarly, the survival rates of the CP and CP + SOD groups were similar, indicating that CP does not produce O_2_^•−^. However, due to the acid-induced in the TME, CNP released of NCTD and Cu^2+^/Cu^+^, CP released of Cu^2+^/Cu^+^. Compared to treating by CNP, the cell activity treating by CNP + SOD increased and was similar that of the treatment of CP + SOD, indicating that CNP can produce some O_2_^•−^ and affect cell activity. The changes in intracellular ROS levels were detected using DCFH-DA (a kind of ROS fluorescent probe). Similar results were obtained, showing that CNP produced the maximum green fluorescence, indicating its strong ROS generation ability (Fig. 6e and S15). Furthermore, we detected the intracellular generation of ·OH, ^1^O_2_ and O_2_^•−^ using the fluorescent probes O26, SOSG, and DHE, respectively. As shown in Fig. 6e, NCTD, CP, and CNP all generate various ROS to a certain extent. This finding is consistent with the trend observed in the extracellular ROS detection.

### 3.4 Anti-tumor effect *in vivo*

**Fig. 7:**
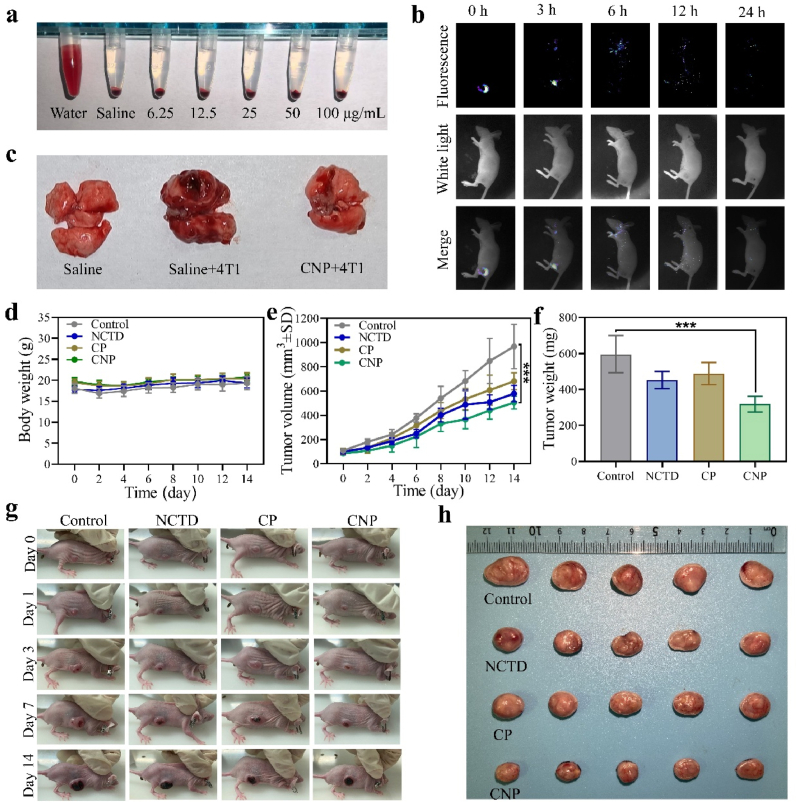
(a) Hemolysis analysis of blood incubated with water (positive control), Saline (negative control), and the different concentrations of CNP (inset: thecorresponding digital photograph). (b) *In vivo* images of the tumor-bearing mice were administrated with fluorescently labeled CNP. (c) Photographs of the lungs after the lung metastasis inhibition assessment experiment. (d) Weight curves of mice with different treatments in 2 weeks. (e) Tumor volume curves. (f) Tumor weights (∗∗∗p < 0.001). (g) Representative photographs of mice during treatment. (h) Photographs of tumor dissection after treatment.

**Fig. 8:**
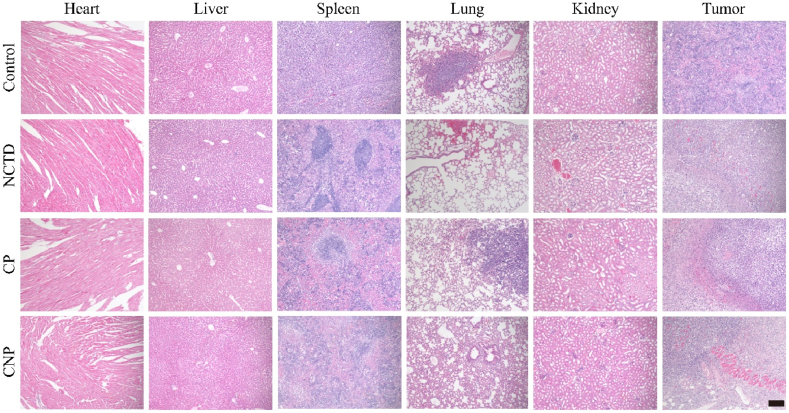
The H&E staining of tumor and organs tissue with different treatments (Scale bar: 200 μm).

Based on the experimental results of effective CT and CDT synergistic treatment with the nanocatalyst in vitro, we used a 4T1 tumor-bearing mouse model to studied the therapeutic effect of CNP. Hemolysis analysis indicated that minimal hemolytic red blood cells were observed, even when CNP were present at a concentration of 100 μg/mL (Fig. 7a) [53]. *In vivo* imaging using fluorescently labeled CNP nanoparticles shown that CNP accumulated at the tumor site in high concentration within 3 h post-injection. Subsequently, influenced by the TME, the nanomedicine and the fluorescent dye were progressively released. This resulted in the fluorescence signal initially spreading systemically and eventually being cleared via metabolic processes (Fig. 7b). Lung metastasis of breast cancer is the most severe type of breast cancer metastasis. Therefore, we established a lung metastasis experimental model by injecting 4T1 cells into the tail vein of mice to explore the lung metastasis inhibitory ability of CNP. The lungs of normal mice were pink and smooth without nodules, while the lungs of the breast cancer model group injected with normal saline were dark red and had significantly more nodules on the surface. However, the number of metastatic nodules on the surface of the lungs of mice treated with CNP was significantly reduced, indicating that CNP has a good inhibitory effect on lung metastasis (Fig. 7c). When the tumor volume of 4T1 tumor-bearing mouse model reached 60–100 mm^3^, the mice were divided into four groups in random: control (Saline), CT treatment (NCTD), CDT treatment (CP), and synergistic treatment (CNP). Over the course of 2 weeks of treatment administration, the body weight and tumor volume of mice were monitored every 2 days. During this period, weight changes in all four groups were insignificant and negligible (Fig. 7d). Compared with the control, the CP treatment had the weakest tumor inhibition ability, followed by the NCTD treatment, and the CNP treatment had the best tumor inhibition ability (Fig. 7e). Similarly, tumor weight of all treatments indicated that CNP had a good therapeutic effect (Fig. 7f). After 2 weeks of treatment, the tumors treating by CNP were significantly smaller than those treating by the Saline (Fig. 7g). Moreover, tumor tissue photographs taken 2 weeks after treatments can also get the same conclusion (Fig. 7h), which were consistent with the trend of the MTT assay results. Additionally, hematoxylin and eosin (H&E) staining analysis (Fig. 8) showed that lung tissue sections in the NCTD group were damaged, while major organ sections in the CP and CNP groups showed no significant changes, indicating good biosafety. This suggests that the damage to mice during CNP treatment was negligible. CNP has a good anti-tumor effect and biosafety.

## 4 Conclusion

In summary, we have designed and successfully synthesized a nanocatalyst with NCTD/Cu^2+^ dual-depletion of GSH and pH responsiveness for CT/CDT synergistic tumor therapy. Upon reaching the tumor, due to the weakly acidic TME, the catalyst releases Chinese medicine active molecule NCTD and Cu^2+^ in a pH-responsive manner. Both components consume GSH, and respectively generate ROS, reducing the antioxidant capacity of cancer cells while increasing oxidative stress. This disrupts cellular redox homeostasis, leading to mitochondrial dysfunction and inducing tumor cell apoptosis. Furthermore, in the breast cancer tumor model, CNP can effectively inhibit tumor growth without significant systemic toxicity, achieving high-efficiency synergistic treatment of CT and CDT. Thus, this study provides a highly efficient nanocatalyst for the synergistic therapy of CT and CDT in breast cancer cells and provides an example of designing a nanocatalyst capable of dual GSH depletion and pH responsiveness.

## CRediT authorship contribution statement

**Xiaohuan Guo:** Writing – original draft, Validation, Methodology, Investigation, Data curation, Conceptualization. **Bingbing Cai:** Validation, Investigation, Data curation. **Qi Fang:** Visualization, Data curation. **Yanyan Chen:** Formal analysis, Conceptualization. **Yuzhu Zhou:** Methodology, Investigation. **Zhixing Liang:** Investigation, Formal analysis. **Changchun Wen:** Writing – review & editing, Project administration, Investigation, Funding acquisition. **Yan-Cheng Liu:** Supervision, Methodology, Funding acquisition. **Hong Liang:** Supervision, Methodology, Funding acquisition.

## Declaration of competing interest

The authors declare that they have no known competing financial interests or personal relationships that could have appeared to influence the work reported in this paper.

## Data Availability

Data will be made available on request.
