# Supplementary material for: Norcantharidin/Cu2+ dual-depleting GSH nanocatalyst with pH-responsive for CT/CDT synergistic cancer therapy
Source: Mater Today Bio. 2025 Jun 6;33:101959. doi: 10.1016/j.mtbio.2025.101959 (PMC12180979; doi:10.1016/j.mtbio.2025.101959)
Supplement: Multimedia component 1 [file mmc1.docx]

**Supporting Information**

**Norcantharidin/Cu^2+^ dual-depleting GSH nanocatalyst with pH-responsive for CT/CDT synergistic cancer therapy**

Xiaohuan Guo^1^, Bingbing Cai^1^, Qi Fang, Yanyan Chen, Yuzhu Zhou, Zhixing Liang, Changchun Wen*, Yan-Cheng Liu*, Hong Liang*

State Key Laboratory for Chemistry and Molecular Engineering of Medicinal Resources, Key Laboratory for Chemistry and Molecular Engineering of Medicinal Resources (Ministry of Education of China), Guangxi Key Laboratory of Chemistry and Molecular Engineering of Medicinal Resources, School of Chemistry and Pharmaceutical Sciences, Guangxi Normal University, Guilin 541004, China


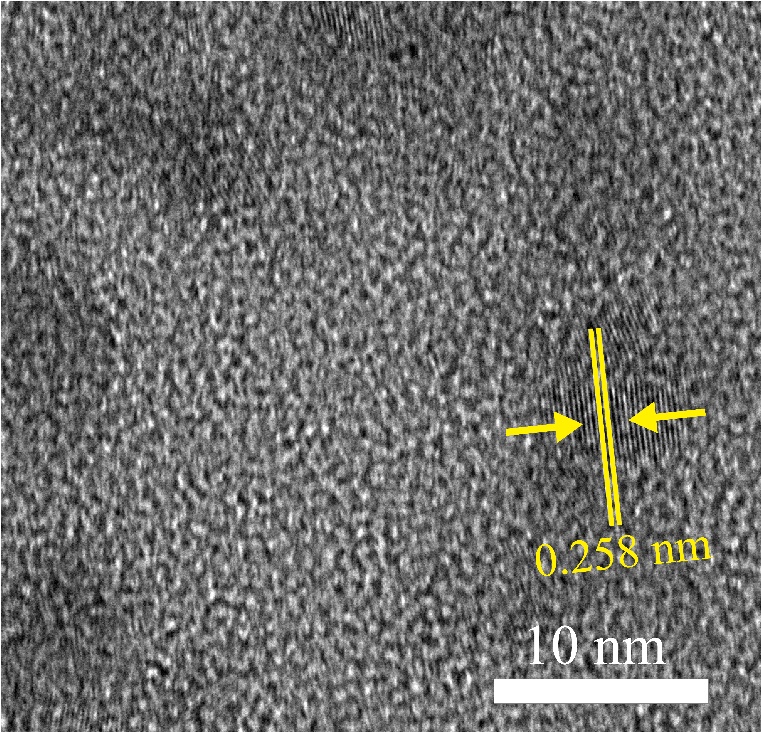


Fig. S1. HRTEM of CNP.


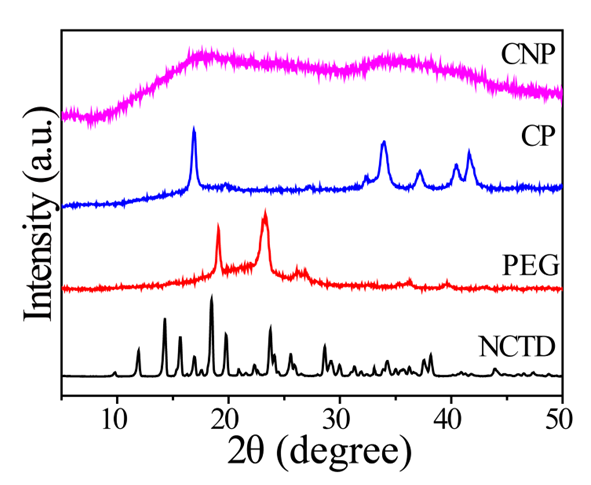


Fig. S2. X-ray diffractometry spectra pattern of NCTD, PEG, CP and CNP.


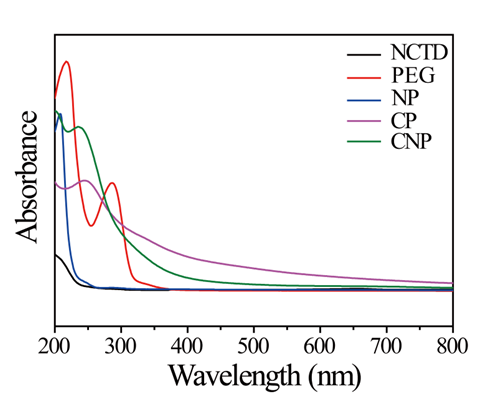


Fig. S3. UV-vis spectra of NCTD, PEG, NP, CP, and CNP.


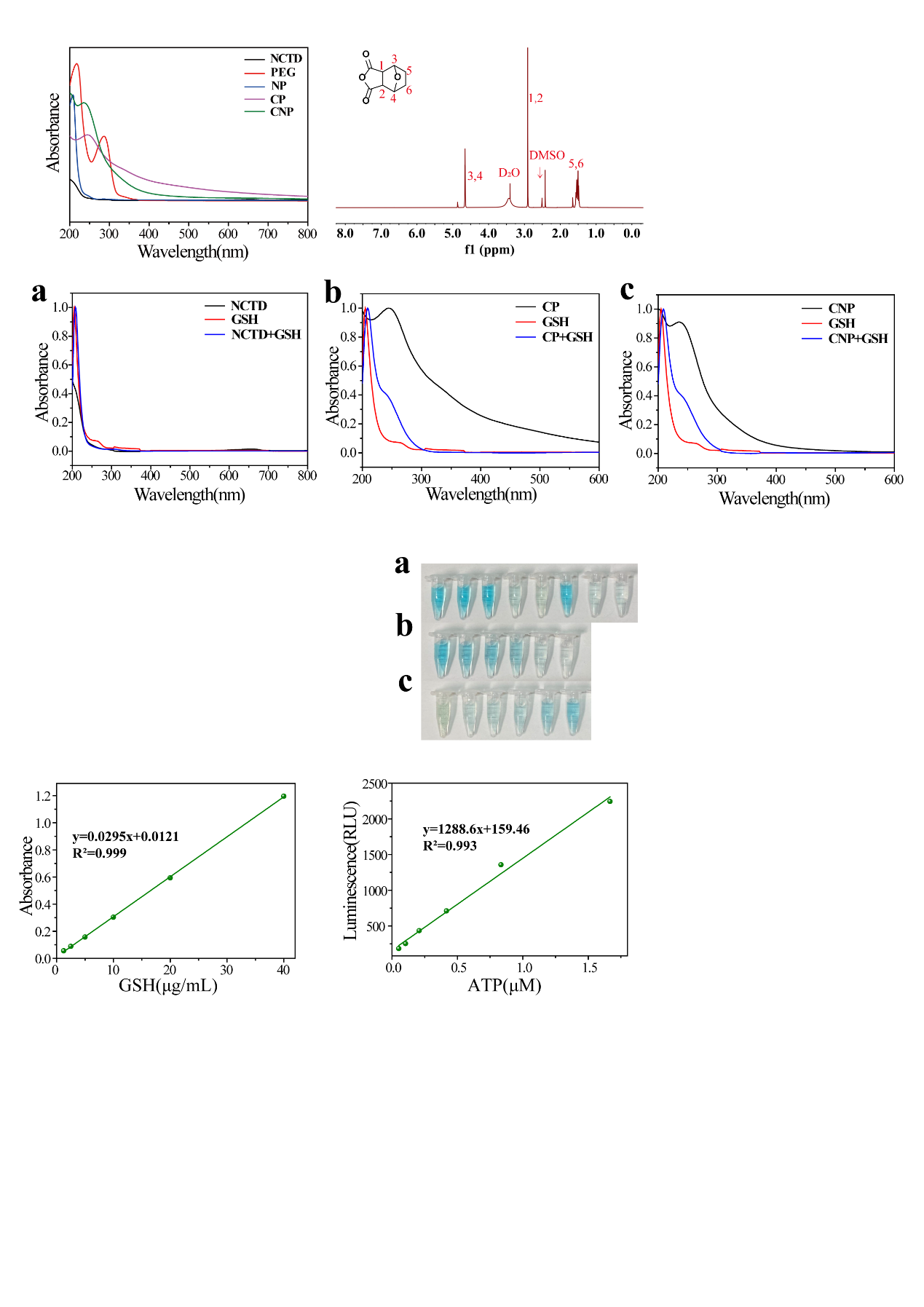


Fig. S4. ^1^H NMR spectrum of NCTD in DMSO.


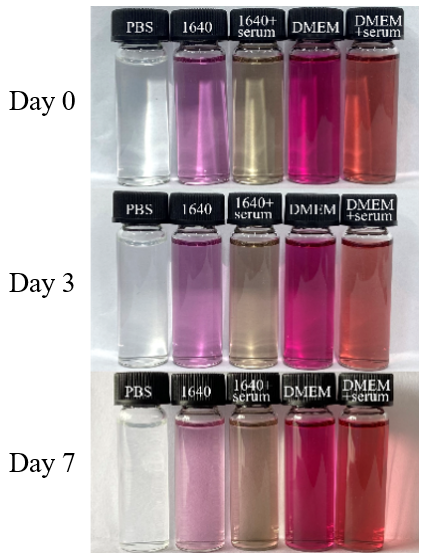


Fig. S5. The water dispersibility and stability of CNP in physiological environment.


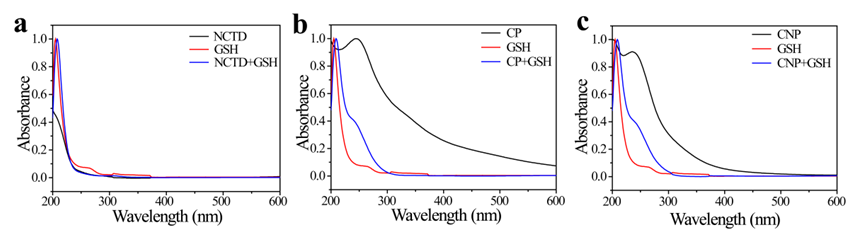


Fig. S6. UV-vis spectra of GSH with NCTD, CP, and CNP.


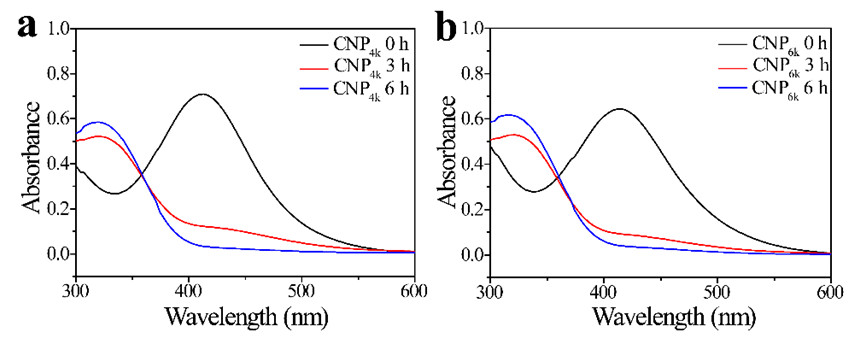


Fig. S7. DTNB experiments of CNP_4k_ and CNP_6k_.


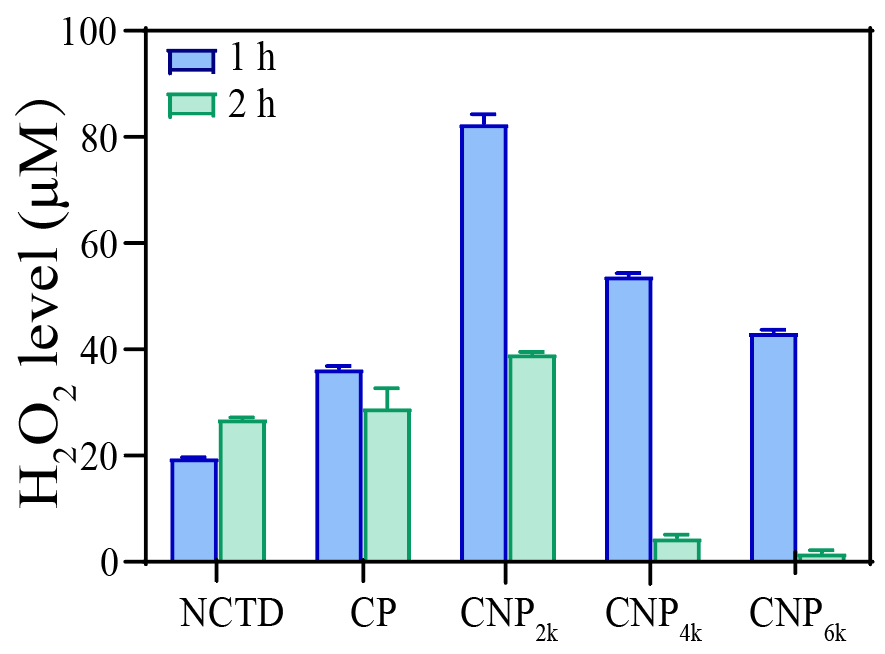


Fig. S8. The H_2_O_2_ contents of NCTD, CP, CNP_2k_, CNP_4k_, CNP_6k_ and GSH after incubation at 37 °C for different time points.


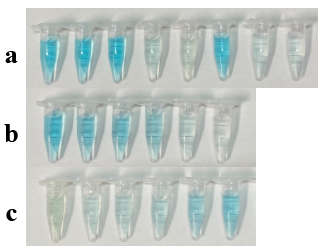


Fig. S9. Figures of degradation ability of MB under different conditions. (a) Add different substrates. (b) Different concentrations of CNP. (c) Different concentrations of GSH.


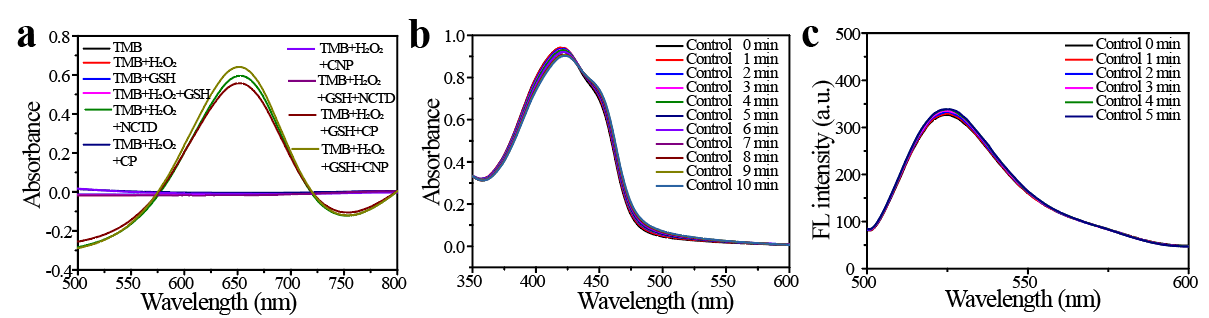


Fig. S10. (a) The generation of ·OH when TMB was incubated with different substrates for 10 min; (b) The generation of ¹O_2_ when DPBF was incubated with H_2_O_2_ for different periods of time; (c) The generation of O_2_^•−^ when DHR123 was incubated with H_2_O_2_ for different periods of time.


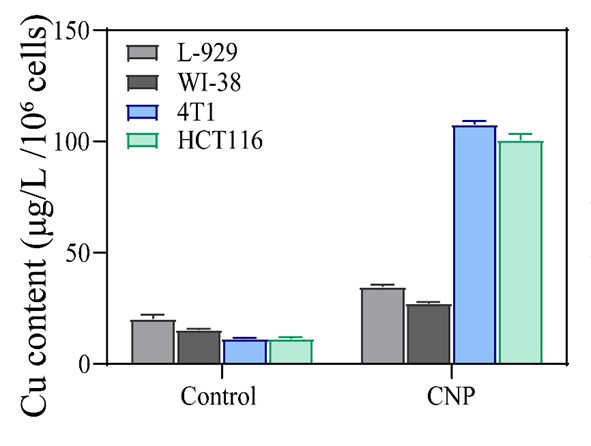


Fig. S11. The concentration of copper of CNP uptake by individual cells.


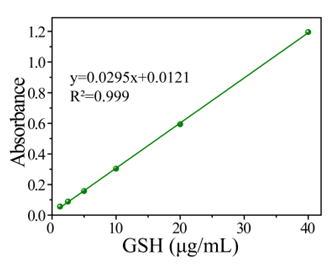


Fig. S12. Standard curve of GSH level determination.


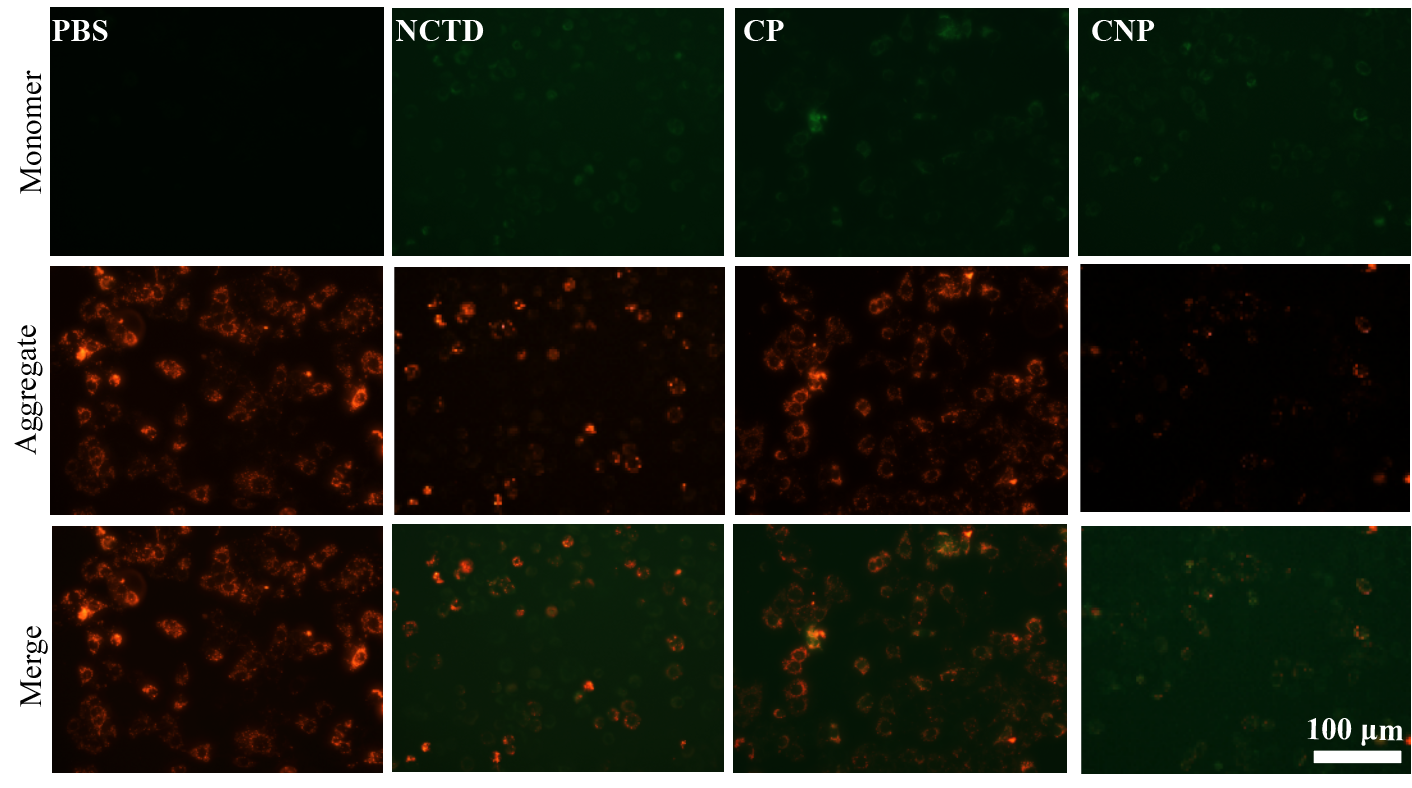


Fig. S13 Mitochondrial membrane potential fluorescence imaging.


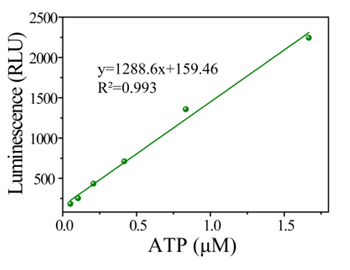


Fig. S14. Standard curve of ATP level determination.


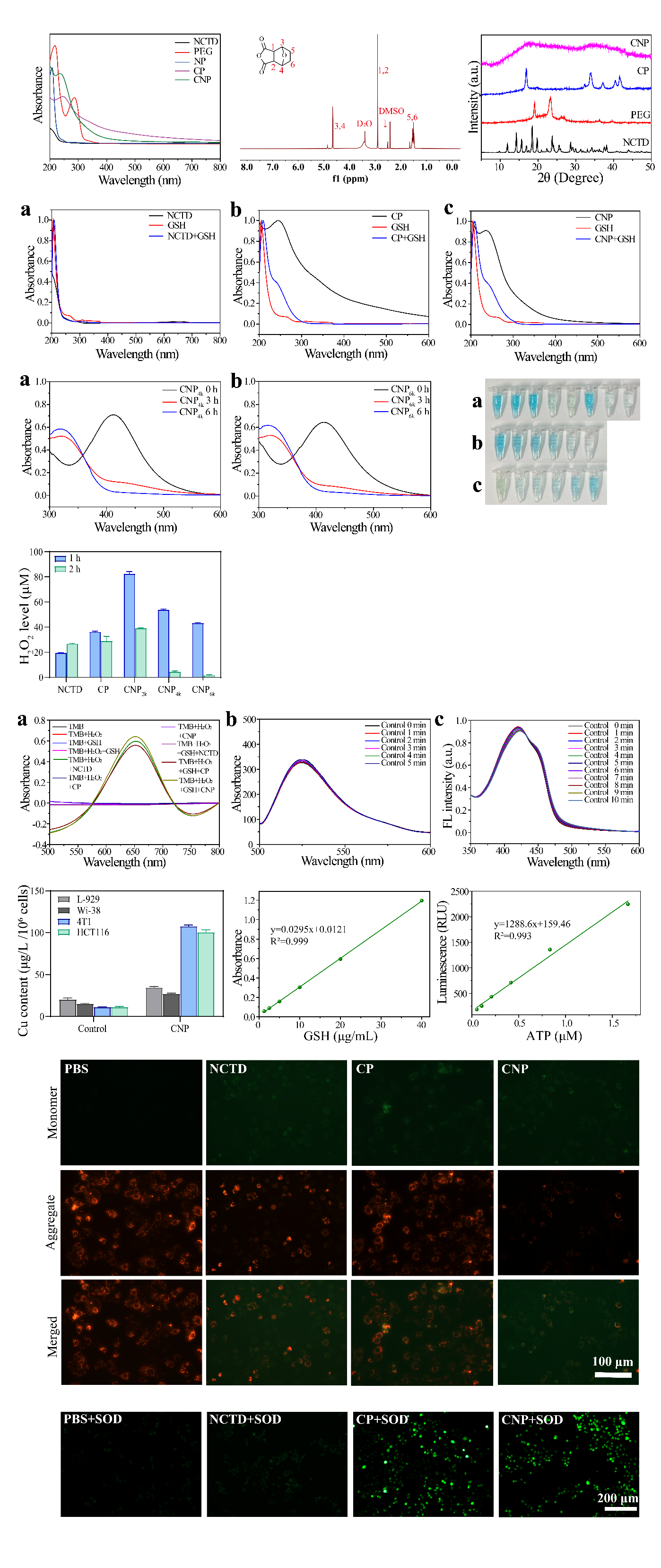


Fig. S15. ROS fluorescence staining after different treatments (green fluorescence).
